# Gastrointestinal symptoms and fecal nucleic acid testing of children with 2019 coronavirus disease: a systematic review and meta-analysis

**DOI:** 10.1038/s41598-020-74913-0

**Published:** 2020-10-20

**Authors:** Ji-gan Wang, Hai-rong Cui, Hua-bo Tang, Xiu-li Deng

**Affiliations:** 1 Maternal and Child Health Hospital of Guangxi Zhuang Autonomous Region, Nanning, 530003 China; 2 The First Affiliated Hospital of Guangxi Medical University, Nanning, 530021 China

**Keywords:** Gastrointestinal system, Paediatrics

## Abstract

In order to understand the clinical manifestations and incidence of gastrointestinal symptoms of coronavirus disease (COVID-19) in children and discuss the importance of fecal nucleic acid testing.We retrospectively analyzed studies on gastrointestinal symptoms and fecal nucleic acid detection in pediatric COVID-19 patients from January 1, 2020 to August 10, 2020, including prospective clinical studies and case reports. The results of fecal nucleic acid detection were analyzed systematically. Stata12.0 software was used for meta-analysis.The results showed that the most common gastrointestinal symptoms in children with COVID-19 were vomiting and diarrhea, with a total incidence of 17.7% (95% Cl 13.9–21.5%). However, the prevalence of gastrointestinal symptoms in other countries (21.1%, 95% CI 16.5–25.7%) was higher compared to China (12.9%, 95% CI 8–17.7%). In Wuhan, the pooled prevalence was much higher (41.3%, 95% CI 3.2–79.4%) compared to areas outside Wuhan in China (7.1%, 95% CI 4.0–10.3%). The positive rate of fecal nucleic acid testing in COVID-19 children was relatively high at 85.8% (91/106). Additionally, 71.2% (52/73) were still positive for fecal nucleic acid after respiratory tract specimens turned negative. One and two weeks after the respiratory tract specimens turned nucleic acid-negative, 45.2% (33/73) and 34.2% (25/73) patients, respectively, remained fecal nucleic acid-positive. The longest interval between the respiratory tract specimens turning negative and fecal specimens turning negative exceeded 70 days. Conclusions and relevance: gastrointestinal symptoms in pediatric COVID-19 are relatively common. Attention should be paid to the detection of fecal nucleic acids in children. Fecal nucleic acid-negative status should be considered as one of the desegregation standards.

## Introduction

Currently, coronavirus disease (COVID-19) has become a global pandemic. There are reports of a large number of cases across the world^1,2^. It mainly infects the middle-aged and elderly, and the mortality rate is the highest in patients with comorbid diseases^3,4^. Therefore, it was believed that children were not easily infected during the early stages of the pandemic. However, with the development of the pandemic, pediatric cases of COVID-19, including severe cases, began to emerge^5^. Consequently, people began to pay attention to the pandemic in children. The main clinical manifestations of COVID-19 are fever, dry cough, and fatigue. Majority of the COVID-19 pediatric patients have nasal obstruction, fever, runny nose, pharyngalgia, muscle pain, and other symptoms^6^. Therefore, there is greater focus on patients with respiratory tract infection symptoms as the presenting symptoms. Since gastrointestinal symptoms are not typical in children, especially as the initial symptoms, they are often ignored. The detection of viral nucleic acid in respiratory tract specimens is the main priority when treating children with COVID-19 in the clinic, while fecal nucleic acid detection is often neglected. Therefore, children with gastrointestinal symptoms as the predominant manifestation of COVID-19 are often misdiagnosed. Currently, there are no large prospective double-blind controlled studies on the gastrointestinal symptoms of COVID-19 and fecal nucleic acid detection in children. Therefore, this study aimed to summarize the gastrointestinal symptoms and fecal nucleic acid detection in children with COVID-19.

PROSPERO registration number: CRD42020190358.

## Materials and methods

### Literature retrieval strategy

PubMed, Web of Science, Embase, Johns Hopkins University published data, as well as the Chinese databases CNKI, Wanfang, and Chongqing Weipu data were searched electronically to collect literature reporting the characteristics of gastrointestinal symptoms of COVID-19 in children, and the retrieval period was from January 1, 2020 to August 10, 2020. Both online database retrieval and manual retrieval were used, and the references included in the literature were traced. Subject words as well as free words were used for retrieval, and adjustments were made according to the characteristics of different databases without any limitations regarding the language, race, and region.

The Pubmed search strategy was as follows

#1 (children) OR (child) OR (kid) OR (pediatric).

#2 (clinical feature) OR (epidemiology) OR (vomiting) OR (diarrhea) OR (stomachache).

#3 (2019-nCoV) OR (COVID-19) OR (SARS-CoV-2) OR (Corona Virus Disease).

#1 AND #2 AND #3

### Literature screening and data extraction

Two researchers independently searched and screened the literature and collected and cross-checked the relevant data. If there was any dispute, it was discussed or resolved with the help of a third researcher.

*Inclusion criteria* 1. Research types: cohort study, case–control study, and case analysis; 2. Subjects: children with COVID-19; 3. Observation index: clinical manifestations of COVID-19 in children including gastrointestinal symptoms such as diarrhea and vomiting.

*Exclusion criteria* 1. Repeated publication of the same research; 2. studies on adults; 3. incomplete or missing data or analysis, and inability to obtain the data literature.

### Study of bias risk assessment

We included a case series study, using the National institute for Clinical Optimization.

Clinical excellence (NICE) for quality evaluation^7^. The evaluation items were as follows: The cases included in the case series should (1) come from different levels of medical institutions that carry out multi-center research; (2) clearly describe the research hypothesis or purpose; (3) have clear exclusion criteria; (4) have a clear definition of the measurement of results; (5) present collected data that achieves the expected purpose; (6) accurately describe that patients are continuously recruited; (7) describe the main findings clearly; (8) analyze and report the results in layers. One point is given for each item, and a total score ≥ 4 out of 8 points is considered as high-quality research. Two researchers independently evaluated the quality and cross-checked the results.

### Statistical analysis

Meta-analysis was performed using the Stata 12.0 software. First, the original ratio (R) was transformed by double arcsine to make it conform to a normal distribution, and then the transform ratio (TR) was analyzed by meta-analysis. Subsequently, the final rate (r) with the 95% confidence interval (CI) were obtained by converting the results with the formula R = (sin[tr/2])^2^. Meta-analysis was carried out by using the random effect model for all studies. The funnel chart was utilized to assess publication bias, and the meta-analysis significance level was designated as α = 0.05.

### Ethics

As this is a systematic review, ethical approval was not required.

## Results

### Gastrointestinal symptoms of COVID-19

**Figure 1:**
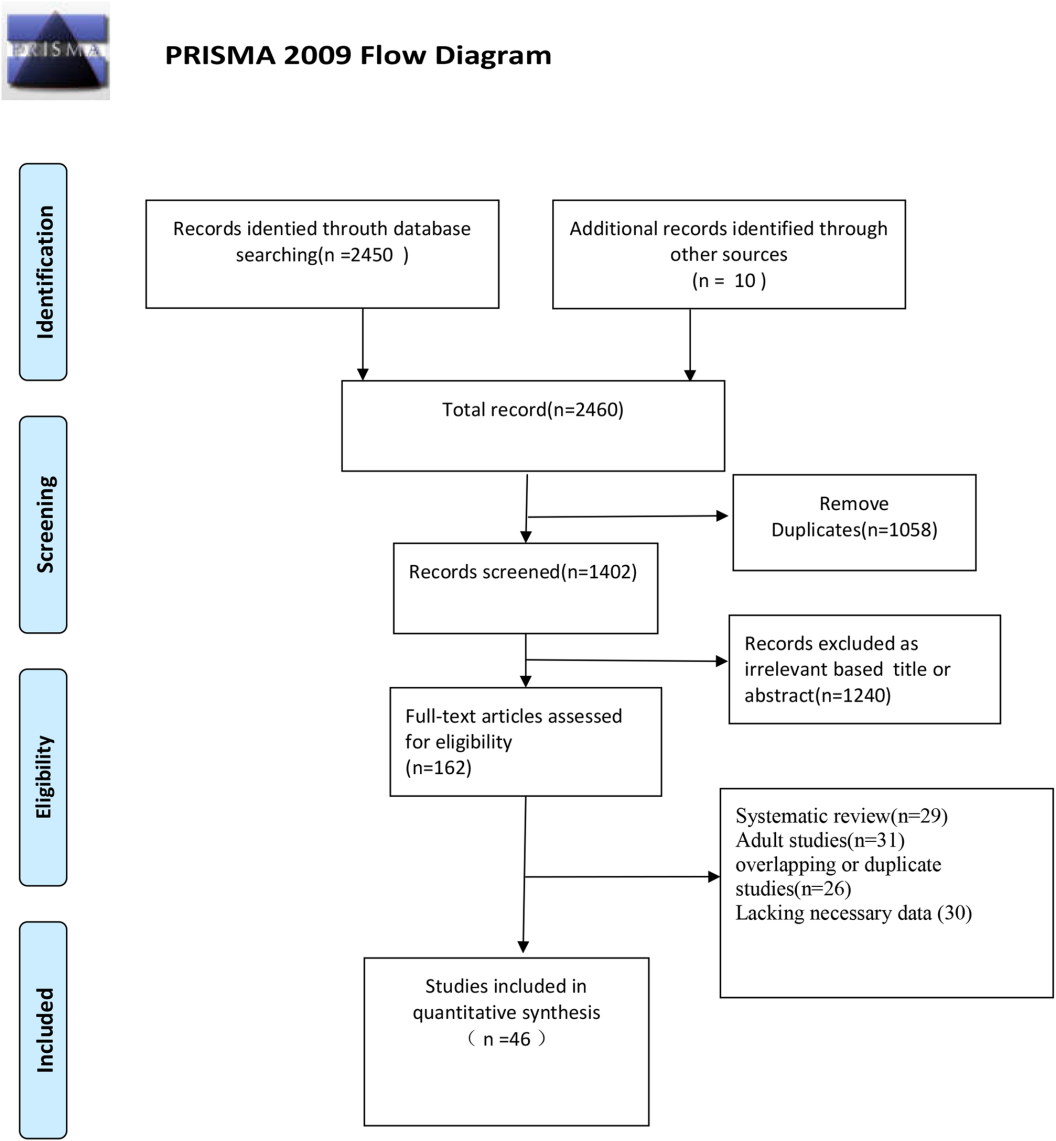
Flow diagram for identification of selected studies in the meta-analysis.

Figure 1 summarizes the article retrieval and abstraction method using the PRISMA guidelines.

**Figure 2:**
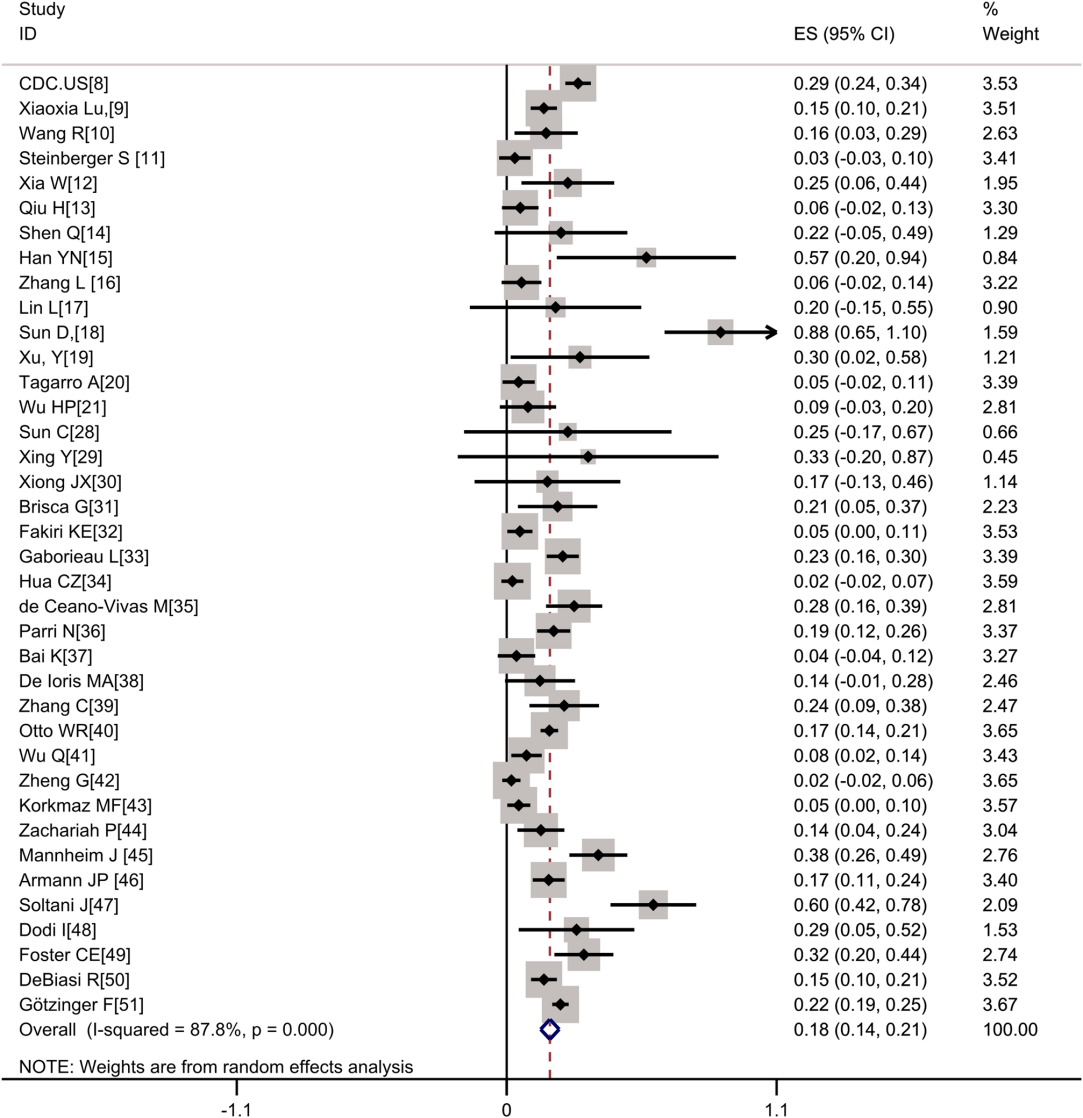
Forest plot of the incidence of gastrointestinal symptoms.

Most research data are concentrated in articles published by China, the United States, and Europe. A total of 46 studies were included in this analysis^8–53^, 38 of which described the gastrointestinal symptoms of patients (Supplementary Table S1). A total of 3028 patients were evaluated in the study, among whom 536 had digestive tract symptoms, accounting for 17.7% (95% CI 13.9–21.5%) of the patients (Fig. 2). Vomiting and diarrhea were the most common gastrointestinal symptoms. When analyzing by country (studies from China versus studies from other countries), the prevalence of gastrointestinal symptoms in countries outside China was 21.1% (95% CI 16.5–25.7%), which was higher than that in China (12.9%, 95% CI 8–17.7%). Among patients in Wuhan, the pooled prevalence was much higher at 41.3% (95% CI 3.2–79.4%) than in areas outside Wuhan in China (7.1%, 95% CI 4.0%–10.3%) (Table 1).

**Table 1:** Subgroup analysis.

Region	Number of included studies	Sample size	Heterogeneity		Effect of the model	Meta-analysis results	
			*P* values	*I*^2^		*R*% (95% CI)	*P* values
China	21	665	0	78.80%	random	12.9% (8–17.7%)	0
Outside China	17	2363	0	86.70%	random	21.1% (16.5–25.7%)	0
Wuhan	3	199	0	94.50%	random	41.3% (3.2–79.4%)	0.034
Outside Wuhan, China	18	466	0.023	44.40%	random	7.1% (4–10.3%)	0
Total	38	3028	0	87.80%	random	17.7% (13.9–21.5%)	0

*CI* confidence interval.

A majority of studies did not describe the stool characteristics or number of bowel movements. Wang Duan et al.^10^ reported that in the six northern provinces of China, patients had bowel movements 2–6 times per day, while Wu Huaping found that in the Jiangxi province of China, the frequency of diarrhea in affected children was 3–4 times per day^21^.

### Subgroup analysis

The heterogeneity of this study was relatively large. To explore the source of heterogeneity, we analyzed according to the region (country or region) where the research object was located. It was discovered that the analysis results of each subgroup were basically consistent with the overall results, and there were no significant differences between the heterogeneity of each subgroup and the overall heterogeneity. Therefore, was considered that the region of the research object was not the main source of heterogeneity (Table 1).

### Fecal testing for viral nucleic acid

Thirteen reports included in the present study described fecal nucleic acid examination (Table 2). The positive rate of fecal nucleic acid testing in COVID-19 patients was 85.8% (91/106). In reports from China^19,27,29,34,41,52^, the positive rate of all stool samples tested was close to or reached 100%. Additionally, 71.2% (52/73) were still positive for fecal nucleic acid after the respiratory tract specimens turned negative, 45.2% (33/73) were fecal nucleic acid-positive one week after the respiratory tract specimen was nucleic acid-negative, and 34.2% (25/73) were fecal nucleic acid-positive two weeks after a respiratory tract nucleic acid negative test. A study from Anhui Province in China found that the longest interval between the respiratory tract specimen turning negative and fecal specimen turning negative exceeded 70 days^34^. However, in a study on three neonates^27^, respiratory tract and fecal nucleic acid tests were positive 2 and 4 days after birth, respectively, and the fecal and respiratory tract specimens were negative on the 6th day after birth.

**Table 2:** Stool RT-PCR test of SARS-COV-2 infected patients.

Authors	Time period of inclusion in the study	Region	Total people	Number of fecal positive results	Age	Clinical picture	Imaging characteristics	The respiratory PCR test was negative, while the stool test was positive	Time difference between fecal NEGATIVE PCR and respiratory tract negative PCR (D)
Park JY^22^	February 18, 2020	Korea	1	1	10 years	Low heat, little sputum	The CT findings are mild pneumonia	0	> 1
Cui Y^23^	January 28, 2020	Kweichow, China	1	1	55 days	Runny nose and dry cough	Flaky shadows and ground-glass opacity	0	+ 18
Zhang YH^24^	January 26, 2020	Haikou city, China	1	1	3 month	Fever	There seems to be a small amount of patchy shadow in the right lower lung field	0	+ 1
Cai J^25^	19 January to 3 February 2020	Shanghai, China	10	6 (another 4 cases of fecal nucleic acid were not tested)	Median 74 m(range 3–131 m)	Symptoms of respiratory tract infection	Unilateral patchy infiltration occurred in 4 of the 10 patients	0	> 18 − 12 > 12 > 11 > 12 > 15 All of them were discharged from hospital on February 19
Zeng LK^26^	February 5, 2020	Wuhan Children's Hospital	1	1	17 days	Sneezing, vomiting milk	Small bands of fuzzy shadows are seen in both lung fields on CT	0	> 1
Xu Y^19^	22 January to 20 February 2020	Guangzhou Women and Children Medical Center	10	8	2 month–15 years	Fever, cough, diarrhea	Isolated or patchy hyaline opacity occurred in 5 patients	0	+ 19 + 1 > 18 + 5 > 18 > 19 + 4 > 3
Zeng L^27^	January to February 2020	Wuhan Children's Hospital	3	3	1 days	All had fever and pneumonia	Chest radiographs suggested pneumonia	0	0
Xing Y^29^	17 January to 23 February 2020	Qingdao Maternal and Child Health Care Hospital, Qingdao, China	3	3	NA	Mild fever	NA	NA	+ 8 + 20 + 20
Hua CZ^34^	By 29 February 2020	Zhejiang province, China	35	32	From 3 months 20 days to 14 years with a mean of 8.16 years (SD: 4.07)	One had diarrhea, others were fever, cough and asymptomatic	Pneumonia or no abnormality	NA	18 > 1d 14 > 7d 12 > 14d 1 > 70d
De Ioris MA^38^	16 March 2020 to 8 April 2020	Bambino Gesù Children Hospital, Rome, Italy	22	15	Median age was 84 months (range, 8 days to 210 months)	All were characterized by fever and diarrhea	NA	NA	NA
Wu Q^41^	January 20 to February 27 of 2020	Qingdao Women and Children’s Hospital and Wuhan Children’s Hospital. China	10	10	median (range) 6.00 (0.10–15.08)	Diarrhea 3 Anorexia 3	NA	NA	8 > 1d 2 > 23d median of 11 days
Zhang B^52^	Feb1-May7	First Affiliated Hospital of Jinan University and Dongguan Ninth People's Hospital	3	3	14 years 13 years 10 month	Asymptomatic Asymptomatic Pneumonia	Pneumonia	+ 1 + 1	NA
Du W^53^	January 23, 2020 to March 9, 2020	Jinan Infectious Disease Hospital Affiliated to Shandong University	10	7	Median age of 5.08 years (range, 9 month-14 years)	NA	NA	NA	+ 8 + 17 + 13 + 17 Na Na NA

### Publication bias

**Figure 3:**
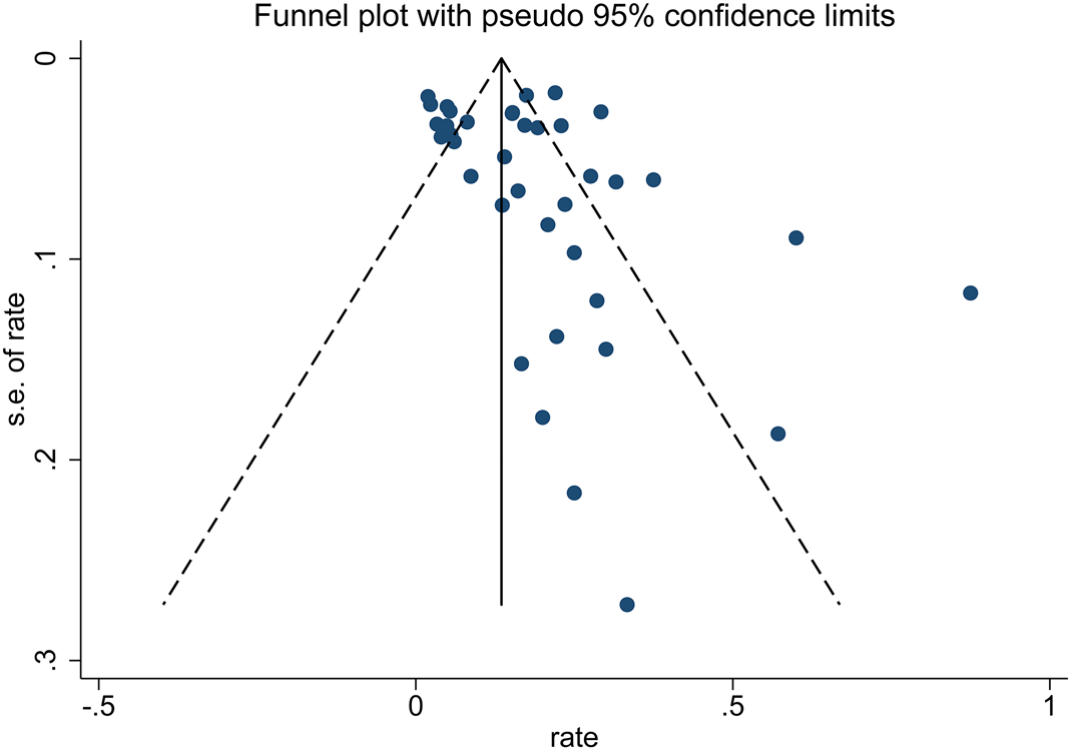
Funnel plot assessing publication bias.

The funnel plot (Fig. 3) shows the presence of a possible publication bias. Most of the research quality scores were not high. Our confidence in the pooled estimates of prevalence was reduced because of concerns regarding risk of bias (selection bias, detection bias, and attrition bias), heterogeneity of the tested patient populations (inconsistency), as well as issues pertaining to indirectness (the majority of studies included primarily symptomatic hospitalized patients instead of all patients with COVID-19). Additionally, most of the studies were retrospective cohort series and did not specify if consecutive patients were included in the analysis. These factors may have contributed to the heterogeneity of findings across studies.

## Discussion

### Incidence of gastrointestinal symptoms

In the early stages of the pandemic, there was a shared misconception that children were not easily infected^1^. However, with the spread of the pandemic, the number of infected children is increasing and several severe pediatric cases have been reported^48^. It is sometimes difficult to distinguish the gastrointestinal symptoms of pediatric COVID-19 from those caused by another viral illness, side effects of drugs, and digestive tract symptoms such as nausea and diarrhea caused by the disturbance of gastrointestinal flora by the fever itself. Some studies^49^ have found that 20.4% children use antibiotics that cause diarrhea, and the diarrhea is more severe in younger patients with lower respiratory tract infections treated with intravenous antibiotics. Moreover, we discovered that the total incidence of gastrointestinal symptoms in children with COVID-19 was 17.7%; unfortunately, not all the studies described a control group when investigating the incidence of gastrointestinal symptoms in an antibiotic treatment group and non-antibiotic treatment group. In a meta-analysis^50^ of predominantly adult studies, 60 studies (including 4243 patients with COVID-19) were analyzed and the incidence of gastrointestinal symptoms was found to be 17.6%, which is almost equal to 17.7% found in this study. In addition, we discovered that the incidence of gastrointestinal symptoms in other countries (21.1%) was significantly higher than that in China (12.9%). One reason may be that the gastrointestinal symptoms were not paid attention to in the early stage of the epidemic. However, once the literature was published, gastrointestinal symptoms were described in detail.

### Pathogenesis of COVID-19

Regarding the mechanism of infection of the severe acute respiratory syndrome coronavirus 2 (SARS-CoV-2), it is currently believed that the major determinant of SARS-CoV-2 infection is the S protein, which binds to membrane receptors on host cells and mediates the fusion of the virus and cell membrane. Angiotensin converting enzyme 2 (ACE2) is a homolog of ACE and one of the important receptors on the cell membrane of host cells. The interaction between the S protein and ACE2 promotes the invasion of host cells by SARS-CoV-2. The structure of the SARS-CoV-2 S protein is highly similar to that of the SARS coronavirus (SARS-CoV) S protein; however, SARS-CoV-2 S protein binds to ACE2 with a higher affinity than the SARS-CoV S protein, indicating that SARS-CoV-2 possesses a stronger invasion ability^51^. ACE2 can control intestinal inflammation and diarrhea, and the interaction between SARS-CoV-2 and ACE2 may lead to diarrhea^52^. ACE2 is highly expressed in the small intestine, especially in the proximal and distal intestinal epithelial cells; therefore, the small intestine is more vulnerable to SARS-CoV-2 infection. Previous investigations may have underestimated the incidence of diarrhea among those infected with SARS-CoV-2. Further research is needed to determine whether diarrhea has diagnostic value for SARS-CoV-2. In case of the Middle East Respiratory Syndrome coronavirus (MERS-CoV), which is highly homologous to SARS-CoV-2, it is believed that the intestinal tract is another route of infection and the incidence rate of diarrhea is 20–25%^53^.

### Pathological examination

Till date, there have been no endoscopic and pathologic studies of the digestive tract in pediatric COVID-19 cases. However, a study in adults^54^ demonstrated that there was no obvious damage to the mucosal epithelium of the esophagus, stomach, duodenum, and rectum. In the inherent layers of the stomach, duodenum, and rectum, a large number of infiltrating plasma cells and lymphocytes were seen accompanied by interstitial edema. ACE2, the virus host receptor, is mainly found in the cytoplasm of gastrointestinal epithelial cells and virus nucleocapsid proteins were found in the cytoplasm of duodenal and rectal glandular epithelial cells.

### Positive rate and significance of fecal nucleic acids

In a recent study^54^ on 73 hospitalized adult patients in China, the feces of 53.42% of the patients were positive for the viral RNA, the duration for which positive fecal results were obtained ranged from 1 to 12 days, and 23.29% of the patients were still fecal nucleic acid-positive after being confirmed respiratory nucleic acid-negative. Compared with adults, the present study found that the nucleic acid positivity rate of feces in children was higher (85.8%). A study reported that among 59 patients with COVID-19 in Hong Kong, 15 (25.4%) had gastrointestinal symptoms and nine (15.3%) had positive stool viral RNA test results. The detection rates of fecal viral RNA were 38.5% and 8.7% in people with and without diarrhea, respectively^50^. At present, there is no relevant study on whether there is a difference in the positive rate of fecal nucleic acid testing in COVID-19 children with and without diarrhea.

In a recent study conducted from January 16, 2020 to February 8, 2020, the Chinese CDC reported 2135 pediatric COVID-19 patients (including confirmed and suspected cases), 94 of whom were asymptomatic (4.4%)^55^. However, a recent study from New York^56^ claimed that 29 (87.9%) out of 33 pregnant women who tested positive for SARS-CoV-2 on admission did not have symptoms of COVID-19 at the time of treatment. This is very worrying data, because it shows that there are more asymptomatic than symptomatic patients; therefore, controlling asymptomatic patients is the key to controlling the pandemic. In children with asymptomatic COVID-19, there is no relevant study on whether the nucleic acid sensitivity of respiratory specimens is higher than that of feces. Furthermore, it remains unknown whether the children in whom the symptoms have resolved and respiratory tract specimens are negative while the stool samples remain positive for viral nucleic acids, are asymptomatic infectious sources. Consequently, it is important to recommend that after recovery and discharge, pediatric patients be isolated at home for more than 2 weeks.

### Prognosis of COVID-19 children with gastrointestinal symptoms

In terms of prognosis, a retrospective comparative study was carried out in patients over 18 years old in the United States^57^. The experimental group included 278 patients with fever and cough due to COVID-19, and the control group included 238 patients with fever and cough attributable to a common respiratory tract infection. The incidence of gastrointestinal symptoms in the two groups was 34.8% and 26.4%, respectively (P = 0.04). In the 278 patients with COVID-19, the course of gastrointestinal symptoms was longer, but the mortality rate and rate of severe disease were lower in patients with gastrointestinal symptoms than in those without such symptoms. At present, there is no prognostic study on children with COVID-19.

### Prevention and treatment

Transmission through respiratory droplets and contact are currently considered to be the main routes of transmission of COVID-19. Nevertheless, there is now increasing evidence of fecal–oral transmission^58^. In clinical practice, doctors mostly pay attention to the manifestations of respiratory infection in children with COVID-19 such as fever, cough, fatigue, etc. For patients in the gastroenterology department who have no respiratory symptoms, it is recommended to adopt the appointment system and time-division diagnosis and treatment to reduce patient aggregation and avoid cross infections. The clinic should be well-ventilated and disinfection of the clinic should be performed daily at the beginning and end of the clinic. Although gastrointestinal symptoms are often ignored, in children with diarrhea, abdominal pain, nausea, vomiting, and other gastrointestinal symptoms accompanied by a low fever, attention should be paid to their epidemiological history with screening of suspected patients. Nucleic acid examination should be performed using throat swabs and anal tests. In daily life, the risk of transmission can be reduced by good hygiene practices, such as washing hands frequently and closing the toilet lid when flushing.

At present, there is no specific drug for COVID-19. Plasma therapy from convalescent patients is considered for those with severe disease^48^; however, this treatment is controversial^59^. Dexamethasone has now been proven to be a good treatment option for the COVID-19^60^. Diarrhea in COVID-19 patients is mostly self-limiting, and symptomatic treatment such as Montmorillonite powder can be used. For critically ill patients, intestinal microecological regulators may be used to maintain the balance of the intestinal flora and prevent secondary infection by intestinal bacterial translocation.

### Study limitations

The number of studies included in the meta-analysis was relatively small, with a relatively large proportion of case reports. Most studies did not report on the duration of the gastrointestinal symptoms preceding the presentation. Additionally, the number of patients was relatively small and the description of the gastrointestinal tract of children in the included studies was not sufficiently detailed. The heterogeneity is large and subgroup analysis can not find the source of heterogeneity, which will affect the accuracy of the results. Therefore, it is necessary to conduct a large-scale double-blind randomized controlled study and include additional research factors such as stool frequency, stool characteristics, number of patients with gastrointestinal symptoms and positive fecal nucleic acid test results, length of hospitalization of fecal nucleic acid-positive patients, severity of illness, and the interrelation between respiratory tract sample nucleic acid and stool nucleic acid findings.

## Conclusions

Gastrointestinal symptoms in pediatric COVID-19 are relatively common. Attention should be paid to the detection of fecal nucleic acids in children. Especially in high-risk epidemic areas, all children with digestive tract symptoms as the presenting symptoms should be tested for the viral fecal nucleic acid. Fecal viral nucleic acid-negative status should be considered one of the discharge standards.

## Supplementary information

Supplementary Information.
