# Supplementary material for: Gastrointestinal symptoms and fecal nucleic acid testing of children with 2019 coronavirus disease: a systematic review and meta-analysis
Source: Sci Rep. 2020 Oct 20;10:17846. doi: 10.1038/s41598-020-74913-0 (PMC7576139; doi:10.1038/s41598-020-74913-0)
Supplement: Supplementary file 1 — Supplementary Information. [file 41598_2020_74913_MOESM1_ESM.doc]

**Gastrointestinal symptoms and fecal nucleic acid testing of children with 2019 coronavirus disease： a systematic review and meta-analysis**

Ji-gan Wang**^a^*** ；Hai-rong Cui**^b^** ; Hua-bo Tang **^a^**；Xiu-li Deng**^a^**

**^a^ Maternal and Child Health Hospital of Guangxi Zhuang Autonomous Region, Nanning, 530003, China**

**^b^ The First Affiliated Hospital of Guangxi Medical University, Nanning, 530021, China**

**∗Corresponding author**

**Correspondence to Ji-gan Wang（Email: 354713144@qq.com）**

**Conflict of interest：The authors declare no conflicts of interest.**

Table S1: Gastrointestinal manifestations in children with SARS-CoV-2 infection

| Authors | Time period of inclusion in the study | Region | Total people | Number of people with gastrointestinal symptoms | Age | Diarrhea frequency | Patients with severe illness or death included in the study | Other | Quality score |
| --- | --- | --- | --- | --- | --- | --- | --- | --- | --- |
| CDC.US [8] | February 12 to April 2, 2020 | US | 291 | Nausea and vomiting 31  Abdominal pain 17  Diarrhea 37 | <18 years | NA | 0 | NA | 4 |
| Xiaoxia Lu [9] | January 28 to February 26, 2020 | Wuhan Children's Hospital | 171 | Diarrhea 15  Vomiting 11 | Average age was 6 years 7 months | NA | 0 | NA | 7 |
| Wang R [10] | January 25 to February 27, 2020 | 6 provinces in northern China | 31 | Diarrhea 3  Vomiting 2 | Average age was 7 years 1 month (range: 6m-17y) | 2~6次  /d | 0 | Diarrhea was the first symptom in 3 cases and vomiting was the first symptom in 1 case | 8 |
| Steinberger S [11] | January 23 to February 8, 2020 | 6 hospitals in Hubei, China | 30 | Diarrhea 1 | Median age was 10 years (range, 6–15 y) | NA | 0 | NA | 6 |
| Xia W [12] | January 23 to February 8, 2020 | Maternal and Child Health Hospital of Hubei Province, China | 20 | Diarrhea 3  Vomiting 2 | Average age was 6 years 7 months (range: 1d-14y) | NA | 0 | NA | 6 |
| Qiu H [13] | January 17 to March 1, 2020 | Zhejiang, China | 36 | Vomiting, diarrhea 2 | Average age was 8 years 3 months (range: 0-16y) | NA | 0 | NA | 6 |
| Shen Q [14] | January 8 to February 19, 2020 | Changsha, China | 9 | Diarrhea 2 | Range: 1-12 years | NA | 0 | NA | 4 |
| Han YN [15] | January 31 to February 16, 2020 | Xi'an, China | 7 | Diarrhea or vomiting 4 | Average age was 1 year 3 months (range: 2m-13y ) | NA | 0 | NA | 5 |
| Zhang L [16] | by February 16，2020 | Anhui Province of China | 33 | Diarrhea, vomiting 2 | 9.59+5.12 years | NA | 0 | NA | 5 |
| Lin L [17] | January 17 to February 15, 2020 | The Fifth Affiliated Hospital of Sun Yat-sen University, Guangdong, China | 5 | Digestive symptoms 1 | <15 years | NA | 0 | NA | 5 |
| Sun D [18] | January 24 to February 24, 2020 | Wuhan Children's Hospital, China | 8 | Nausea and vomiting 4,  Diarrhea 3  Constipation 1 | Range: 2m-15y | NA | All 8 patients were in ICU and there was no death | NA | 4 |
| Xu Y [19] | January 22 to February 20, 2020 | Guangzhou Women and Children's Medical Center, China | 10 | Diarrhea 3 | Range: 2m-15y | NA | 0 | Eight anal tests were positive | 5 |
| Tagarro A [20] | March 2 to March 16, 2020 | Spain | 41 | Gastroenteritis or vomiting 2 | Median age 1 year (range: 3 months-8 years 5 months) | NA | 0 | NA | 4 |
| WuHP [21] | January 21 to February 29, 2020 | Jiangxi, China | 23 | Diarrhea 2 | Median age of 5 years 7 months (range: 3 months-17 years 8 months) | 3-4 times/day | 0 | NA | 5 |
| Sun C [28] | January 24 to February 16, 2020 | Nanyang, China | 4 | Nausea, vomiting, diarrhea 1 | Median age was 6 years (range: 3y‐16 y) | NA | 0 | NA | 4 |
| Xing Y [29] | January 17 to February 23, 2020 | Qingdao Maternity and Child Health Hospital, China | 3 | Gastrointestinal manifestations 1 | NA | NA | 0 | NA | 4 |
| Xiong JX [30] | February 2 to February 15, 2020 | Chongqing, China | 6 | Diarrhea 1 | Range: 8m-13y | NA | 0 | NA | 4 |
| Brisca G [31] | February 24 to April 16，2020 | IRCCS Gaslini Children's Hospital, Genoa, Italy | 24 | Vomiting 2  Diarrhea 3 | Median age was 6.9 years (range: 14 d‐18 y) | NA | 0 | NA | 4 |
| Fakiri KE [32] | March 2 to April 1, 2020 | Mohammad VI university hospital of Marrakesh. Moroccan | 74 | Diarrhea 4 | Median age was 7 y (range: 2 m – 17 y) | NA | 0 | NA | 8 |
| Gaborieau L [33] | 23 March, 2020 to 10 May, 2020 | Suburbs of Paris | 157 | Diarrhea 24  Vomiting 12 | Median age was 1 year (IQR: 0.125-11m) | NA | 16 Patients had severe disease, 3 patients died | NA | 7 |
| Hua CZ [34] | By February 29, 2020 | Zhejiang province, China | 43 | Diarrhea 1 | From 3 months 20 days to 14 years with a mean of 8.16 years (SD: 4.07). | NA | 0 | Feces were tested for SARS‐CoV‐2 RNA in 35 children | 4 |
| de Ceano-Vivas M [35] | March 11 to April 9，2020 | Hospital La Paz (Madrid) | 58 | Diarrhea 7  Vomiting 9 | Median age 35.5 months (IQR) (3.3–146m) | NA | 5 Patients had severe disease | NA | 7 |
| Parri N [36] | February 3 to March 26， 2020 | 10 regions in Italy | 130 | Vomiting 15  Diarrhea 10 | Median age 6 years (0–11y) | NA | 9 Patients had severe disease | NA | 5 |
| Bai K[37] | January 19 to March 12, 2020 | 4 government-designated hospitals in Chongqing | 25 | Diarrhea 1 | Median age was 11.0 years (IQR: 6.3–14.5y) | NA | 0 | NA | 6 |
| De Ioris MA[38] | March16 to April 8，2020 | Bambino Gesù Children Hospital, Rome, Italy | 22 | Diarrhea and vomiting 3 | Median age was 84 months (range, 8 days to 210 months) | NA | 0 | 15 were positive on anal test | 8 |
| Zhang C[39] | January 27 to February 23, 2020 | 4 hospitals in West China | 34 | Diarrhea 4  Vomiting 4 | Median age was 33 months (IQR: 10.00–94.25m) | NA | 0 | NA | 7 |
| Otto WR [40] | March 9 to June 1， 2020 | Children’s Hospital of Philadelphia | 424 | Gastrointestinal symptoms 74 | Median age5.9y[IQR], 1.7–13.2y | NA | 12 Patients had severe disease, 2 people died | NA | 7 |
| Wu Q [41] | January 20 to February 27 ，2020 | Qingdao Women and Children’s Hospital and Wuhan Children’s Hospital.China | 74 | Diarrhea 3  Anorexia 3 | Median 6.y (range: 0.10–15.08y) | NA | Severe pneumonia | NA | 8 |
| Zheng G [42] | January 21 to February 29，2020 | 3 provinces of South China | 52 | Diarrhea 1 | Median age was 9 years (IQR: 4‐12y) | NA | 0 | NA | 7 |
| Korkmaz MF [43] | March 5 to May 5, 2020, | Bursa City Hospital, Turkey | 81 | Vomiting, diarrhea 4 | Median age was 9.50 years (3.16–15.08 years) | NA | 0 | NA | 7 |
| Zachariah P [44] | March 1 to April 15, 2020 | NewYork-Presbyterian Morgan Stanley Children’s Hospital | 50 | Gastrointestinal tract symptoms 7 | 9 years（6d  -18y） | NA | 9 Patients had severe disease | NA | 7 |
| Mannheim J [45] | March 5 to April 8, 2020 | The Chicago Department of Public Health | 64 | Diarrhea 10   Nausea/vomiting 6  Abdominal pain 8 | Median 11 (IQR:7–16y) | NA | 7 Patients had severe disease | NA | 5 |
| Armann JP [46] | March 18 to May 4，2020 | The German Society for Pediatric Infectious Disease | 128 | Gastrointestinal symptoms 22 | NA | na | NA | NA | 5 |
| Soltani J [47] | March 1 to April 15, 2020 | West of Iran | 30 | Diarrhea 3  Nausea 8  Vomiting 7 | NA | NA | 1 patient died | NA | 4 |
| Dodi I [48] | February 26 to April 11, 2020 | “Barilla Children’s Hospital” of Parma | 14 | Diarrhea 2  Vomiting 2 | 0-15 years | NA | 0 | NA | 5 |
| Foster CE [49] | March 10 to April 18, 2020 | Texas Children's Hospital, US | 57 | Diarrhea 8  Vomiting 10 | Median age was 10.7 years (range: 0.1–20.2 y) | NA | NA | NA | 6 |
| DeBiasi R [50] | March 15 to April 30，2020 | Children’s National Hospital | 177 | Diarrhea or vomiting 27 | Median age was 9.6 years (range 0.1-34.2 y) | NA | 9 Patients had severe disease | NA | 6 |
| Götzinger F [51] | By May 7, 2020 | 21 European countries | 582 | Gastrointestinal symptoms 128 | Median age was 5·0 years (IQR: 0·5–12·0 y) | NA | NA | NA | 4 |

NA: not mentioned, IQR: interquartile range

**References**

11.Steinberger, S. *et al.* CT Features of Coronavirus Disease (COVID-19) in 30 Pediatric Patients. *AJR Am. J. Roentgenol.*, 1-9, doi:10.2214/ajr.20.23145 (2020).

12.Xia, W. *et al.* Clinical and CT features in pediatric patients with COVID-19 infection: Different points from adults. *Pediatr. Pulmonol.* **55**, 1169-1174, doi:10.1002/ppul.24718 (2020).

13. Qiu, H. *et al.* Clinical and epidemiological features of 36 children with coronavirus disease 2019 (COVID-19) in Zhejiang, China: an observational cohort study. *Lancet Infect. Dis.* **20**, 689-696, doi:10.1016/s1473-3099(20)30198-5 (2020).

14.Shen, Q. *et al.* Novel coronavirus infection in children outside of Wuhan, China. *Pediatr. Pulmonol.* **55**, 1424-1429, doi:10.1002/ppul.24762 (2020).

15. Han, Y. N. et al. A comparative-descriptive analysis of clinical characteristics in 2019-coronavirus-infected children and adults. J. Med. Virol., doi:10.1002/jmv.25835 (2020).

16.Zhang, L. & Huang, S. Clinical Features of 33 Cases in Children Infected With SARS-CoV-2 in Anhui Province, China-A Multi-Center Retrospective Cohort Study. *Front Public Health* **8**, 255, doi:10.3389/fpubh.2020.00255 (2020).

17.Lin, L. et al. Gastrointestinal symptoms of 95 cases with SARS-CoV-2 infection. Gut 69, 997-1001, doi:10.1136/gutjnl-2020-321013 (2020).

18.Sun, D. et al. Clinical features of severe pediatric patients with coronavirus disease 2019 in Wuhan: a single center's observational study. World J. Pediatr. 16, 251-259, doi:10.1007/s12519-020-00354-4 (2020).

19.Xu, Y. *et al.* Characteristics of pediatric SARS-CoV-2 infection and potential evidence for persistent fecal viral shedding. *Nat. Med.* **26**, 502-505, doi:10.1038/s41591-020-0817-4 (2020).

20.Tagarro, A. *et al.* Screening and Severity of Coronavirus Disease 2019 (COVID-19) in Children in Madrid, Spain. *JAMA Pediatr*, doi:10.1001/jamapediatrics.2020.1346 (2020).

21.Wu HP, *et al.* Clinical analysis of 23 cases of COVID-19 in children under 18 years of age in Jiangxi [J]. Chinese Journal of Contemporary Pediatrics,202,22(05):419-424.DOI: 10.7499/j.issn.1008-8830.2003202 (2020).

22.Park, J. Y., Han, M. S., Park, K. U., Kim, J. Y. & Choi, E. H. First Pediatric Case of Coronavirus Disease 2019 in Korea. J. Korean Med. Sci. 35, e124, doi:10.3346/jkms.2020.35.e124 (2020).

23.Cui, Y. *et al.* A 55-Day-Old Female Infant Infected With 2019 Novel Coronavirus Disease: Presenting With Pneumonia, Liver Injury, and Heart Damage. *J. Infect. Dis.* **221**, 1775-1781, doi:10.1093/infdis/jiaa113 (2020).

24.Zhang YH, *et al*. A case of novel coronavirus infection in a three-month-old baby. Chinese Journal of Pediatrics, (03):182-184,DOI: 10.3760/cma.j.issn.0578-1310.2020.03.004(2020)

25.Cai, J. *et al.* A Case Series of children with 2019 novel coronavirus infection: clinical and epidemiological features. *Clin. Infect. Dis.*, doi:10.1093/cid/ciaa198 (2020).

26.Zeng LK, *et al.* The first neonatal COVID-19 in China [J]. Chinese Journal of Pediatrics,202,58 (04): 279-280. DOI: 10.3760/cma.j.cn112140-20200212-00081(2020).

27. Zeng, L. *et al.* Neonatal Early-Onset Infection With SARS-CoV-2 in 33 Neonates Born to Mothers With COVID-19 in Wuhan, China. *JAMA Pediatr* **174**, 722-725, doi:10.1001/jamapediatrics.2020.0878 (2020).

28.Sun S, *et al*. Analysis of clinical characteristics of 150 cases of novel Coronavirus infection in Nanyang City, Henan Province [J]. Chinese Journal of Tuberculosis and Respiratory Medicine,(06):503-508.DOI: 10.3760/cma.j.cn112147-20200224-00168 (2020)

29.Xing, Y. *et al.* Dynamics of faecal SARS-CoV-2 in infected children during the convalescent phase. *J. Infect.* **81**, 318-356, doi:10.1016/j.jinf.2020.03.049 (2020).

30.Xiong JX, *et al.* Children COVID - 19 patients with CT performance [J/OL]. Chongqing medical: 1-4 [2020-08-25].

31.Brisca, G. *et al.* The early experiences of a single tertiary Italian emergency department treating COVID-19 in children. *Acta Paediatr.*, doi:10.1111/apa.15451 (2020).

32.Fakiri, K. E., Nassih, H., Sab, I. A., Draiss, G. & Bouskraoui, M. Epidemiology and Clinical Features of Coronavirus Disease 2019 in Moroccan Children. *Indian Pediatr.* (2020).

33.Gaborieau, L. *et al.* Epidemiology and Clinical Presentation of Children Hospitalized with SARS-CoV-2 Infection in Suburbs of Paris. *J Clin Med* **9**, doi:10.3390/jcm9072227 (2020).

34.Hua, C. Z. *et al.* Epidemiological features and viral shedding in children with SARS-CoV-2 infection. *J. Med. Virol.*, doi:10.1002/jmv.26180 (2020).

35.de Ceano-Vivas, M. *et al.* SARS-CoV-2 infection in ambulatory and hospitalised Spanish children. *Arch. Dis. Child.* **105**, 808-809, doi:10.1136/archdischild-2020-319366 (2020).

36.Parri, N. *et al.* Characteristic of COVID-19 infection in pediatric patients: early findings from two Italian Pediatric Research Networks. *Eur. J. Pediatr.* **179**, 1315-1323, doi:10.1007/s00431-020-03683-8 (2020).

37. Bai, K. *et al.* Clinical Analysis of 25 COVID-19 Infections in Children. *Pediatr. Infect. Dis. J.* **39**, e100-e103, doi:10.1097/inf.0000000000002740 (2020)..

38. De Ioris, M. A. *et al.* Dynamic Viral Severe Acute Respiratory Syndrome Coronavirus 2 RNA Shedding in Children: Preliminary Data and Clinical Consideration from a Italian Regional Center. *J Pediatric Infect Dis Soc* **9**, 366-369, doi:10.1093/jpids/piaa065 (2020).

39.Zhang, C. *et al.* Clinical and epidemiological characteristics of pediatric SARS-CoV-2 infections in China: A multicenter case series. *PLoS Med.* **17**, e1003130, doi:10.1371/journal.pmed.1003130 (2020).

40. Otto, W. R. et al. The Epidemiology of SARS-CoV-2 in a Pediatric Healthcare Network in the United States. J Pediatric Infect Dis Soc, doi:10.1093/jpids/piaa074 (2020).

41.Wu, Q. et al. Coinfection and Other Clinical Characteristics of COVID-19 in Children. Pediatrics 146, doi:10.1542/peds.2020-0961 (2020).

42.Zheng, G. *et al.* Clinical characteristics of acute respiratory syndrome with SARS-CoV-2 infection in children in South China. *Pediatr. Pulmonol.*, doi:10.1002/ppul.24921 (2020).

43.Korkmaz, M. F., Türe, E., Dorum, B. A. & Kılıç, Z. B. The Epidemiological and Clinical Characteristics of 81 Children with COVID-19 in a Pandemic Hospital in Turkey: an Observational Cohort Study. *J. Korean Med. Sci.* **35**, e236, doi:10.3346/jkms.2020.35.e236 (2020)..

44. Zachariah, P. et al. Epidemiology, Clinical Features, and Disease Severity in Patients With Coronavirus Disease 2019 (COVID-19) in a Children's Hospital in New York City, New York. JAMA Pediatr, e202430, doi:10.1001/jamapediatrics.2020.2430 (2020).

45.Mannheim, J., Gretsch, S., Layden, J. E. & Fricchione, M. J. Characteristics of Hospitalized Pediatric COVID-19 Cases - Chicago, Illinois, March - April 2020. J Pediatric Infect Dis Soc, doi:10.1093/jpids/piaa070 (2020).

46.Armann, J. P. *et al.* Hospital Admission in Children and Adolescents With COVID-19. *Dtsch Arztebl Int* **117**, 373-374, doi:10.3238/arztebl.2020.0373 (2020).

47.Soltani, J. *et al.* Pediatric coronavirus disease 2019 (COVID-19): An insight from west of Iran. *North Clin Istanb* **7**, 284-291, doi:10.14744/nci.2020.90277 (2020).

48.Pathak, E. B., Salemi, J. L., Sobers, N., Menard, J. & Hambleton, I. R. COVID-19 in Children in the United States: Intensive Care Admissions, Estimated Total Infected, and Projected Numbers of Severe Pediatric Cases in 2020. *J. Public Health Manag. Pract.* **26**, 325-333, doi:10.1097/phh.0000000000001190 (2020).

49.Baù, M. *et al.* Risk and Protective Factors for Gastrointestinal Symptoms associated with Antibiotic Treatment in Children: A Population Study. *Pediatr Gastroenterol Hepatol Nutr* **23**, 35-48, doi:10.5223/pghn.2020.23.1.35 (2020).

50.Cheung, K. S. *et al.* Gastrointestinal Manifestations of SARS-CoV-2 Infection and Virus Load in Fecal Samples From a Hong Kong Cohort: Systematic Review and Meta-analysis. *Gastroenterology* **159**, 81-95, doi:10.1053/j.gastro.2020.03.065 (2020).

51.Zhang, X., Li, S. & Niu, S. ACE2 and COVID-19 and the resulting ARDS. *Postgrad. Med. J.* **96**, 403-407, doi:10.1136/postgradmedj-2020-137935 (2020).
